# Associations of changes in body mass index with all-cause and cardiovascular mortality in healthy middle-aged adults

**DOI:** 10.1371/journal.pone.0189180

**Published:** 2017-12-07

**Authors:** In-Jeong Cho, Hyuk-Jae Chang, Ji Min Sung, Young Mi Yun, Hyeon Chang Kim, Namsik Chung

**Affiliations:** 1 Division of Cardiology, Severance Cardiovascular Hospital, Yonsei University College of Medicine, Seoul, Republic of Korea; 2 Severance Biomedical Science Institute, Yonsei University College of Medicine, Seoul, Republic of Korea; 3 Health-IT Acceleration Platform Technology Innovation Center, Yonsei University College of Medicine, Seoul, Republic of Korea; 4 Department of Preventive Medicine, Yonsei University College of Medicine, Seoul, Republic of Korea; Uppsala Clinical Research Center, SWEDEN

## Abstract

**Background:**

Conflicting data exist regarding the association of body mass index (BMI) changes with all-cause and cardiovascular (CV) mortality. The current study investigated the association between changes in BMI and all-cause, CV, and non-CV mortality in a large cohort of middle-aged adults.

**Methods:**

A total of 379,535 adults over 40 years of age without pre-existing CV disease or cancer at baseline were enrolled to undergo a series of at least three health examinations of biennial intervals. Changes in BMI between baseline, midpoint follow-up, and final health examination during mean 9.3 years were defined according to the pattern of BMI change as follows: stable, sustained gain, sustained loss, and fluctuation. The relationship between BMI change category and mortality was assessed by multivariate Cox regression reporting hazard ratio (HR) with 95% confidence interval (95% CI).

**Results:**

During a mean follow-up of 10.7 years for mortality, 12,378 deaths occurred from all causes, of which 2,114 were CV and 10,264 were non-CV deaths. Sustained BMI gain was associated with the lower risk of all-cause (HR 0.89, 95% CI: 0.83–0.95), CV (HR 0.84, 95% CI 0.72–0.98), and non-CV mortality (HR 0.90, 95% CI 0.84–0.96) compared with stable BMI. Conversely, sustained BMI loss (HR 1.25, 95% CI 1.19–1.32) and fluctuation (HR 1.13, 95% CI 1.08–1.19) displayed a higher risk of all-cause mortality compared with stable BMI, which was mainly attributable to the increase in non-CV mortality.

**Conclusion:**

Sustained BMI gains were associated with reduced risk of all-cause, CV, and non-CV mortality in middle-aged healthy adults.

## Introduction

Lean mass tends to peak in the third to fourth decade of life, followed by a steady decline with advancing age.[1] In contrast, body weight increased up to 60 years of age and subsequently decreased in more than 60% of the population.[2] As a consequence, an accumulation of fat mass occurs in most adults during midlife. These findings are based mainly on Western populations, and it is well known that Asian cohorts tend to differ substantially in body weight and size from their Western counterparts. However, body mass index (BMI) gain during midlife has been also shown in Chinese adults, suggesting the presence of the same weight or BMI gain phenomenon in the Asian population.[3]

Obesity has long been established as a major burden for cardiovascular (CV) disease.[4] By extension, high BMI is known to provoke the development and progression of CV risk factors such as hypertension, dyslipidemia, insulin resistance, and diabetes mellitus, which can eventually lead to coronary heart disease and ischemic stroke.[5,6] The onset of overweight and obesity and their sequelae are well recognized, and it is universally recommended that individuals considered to be overweight or obese should be encouraged to participate in a combination of weight loss and physical activity regimens to offset the risks associated with an unhealthy life.[7]

Recently, however, a few studies have documented improved clinical outcomes for heart failure and other chronic disease conditions among overweight patients or those who present with class I obesity compared with normal weight patients, leading to the notion of an obesity paradox.[8,9] Furthermore, there are conflicting data regarding the association between long-term BMI changes and risk of mortality or development of CV risk factors.[7,10–14] To date, whether BMI gains during midlife are associated with a beneficial or detrimental effect on the risk of all-cause or CV mortality has not been thoroughly studied. The current study therefore set out to assess the relationship, if any, between changes in BMI and all-cause, CV, and non-CV mortality among a large sample of healthy middle-aged adults who underwent regular serial health examinations.

## Materials and methods

### Study population

The National Health Insurance System (NHIS) in South Korea is compulsory, and all citizens in the Republic of Korea are required to participate. As described in detail previously,[15] the NHIS in Republic of Korea oversees the national health examination programs.

The NHIS released NHIS-National Health Screening Cohort (NHIS-HEALS), comprised of 514,795 randomly sampled individuals from population that received NHIS health screening examinations between 2002 and 2003, as basically recommended by biennial intervals (annually for manual workers), and were followed up until 2013. The cohort represents approximately 10% of the source population who underwent NHIS examination between 2002 and 2003, and has been followed up to either the time of the participant’s disqualification of health services due to death or emigration or the end of the study period in 2013. The database contains information regarding the health screening questionnaire and laboratory tests, health insurance type, medical bill details, medical treatment, disease histories, and prescription records.

**Fig 1:**
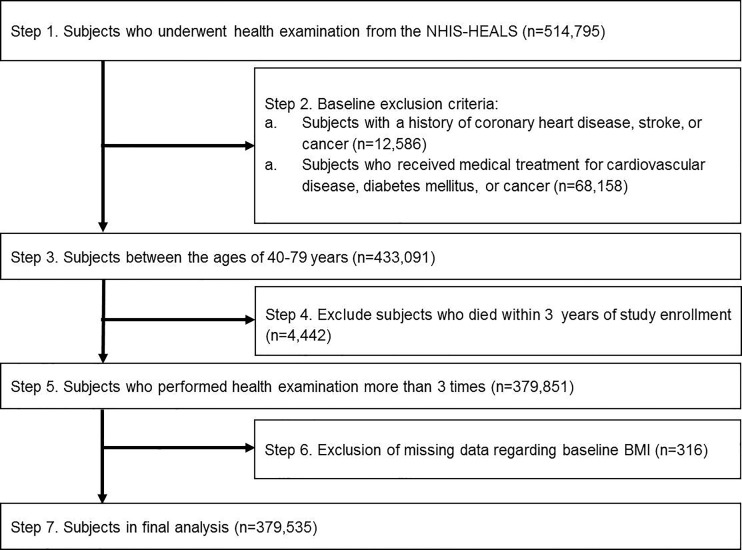
Flow chart of the study population. NHIS-HEALS, National Health Insurance Service-National Health Screening Cohort.

Fig 1 displays the selection criteria for the study population. From the overall cohort of 514,795 subjects who received health examinations between 2002 through 2003, we excluded those with pre-existing histories or treatment history of CV disease, cancer, or cerebrovascular disease. We then selected healthy men and women aged between 40 and 79 years at baseline health examination and excluded those who had died within 3 years following the first examination. We finally selected 379,851 subjects who underwent the health examination more than three times for measurement of BMI variability; patients with missing values for baseline BMI were omitted. In total, 379,535 individuals with more than three repeat measurements for BMI and CV risk factors formed the analytic sample. This study was based on data derived from the NHIS, and informed consent was not specifically obtained from each participant. Data were fully anonymized and de-identified for all analyses. This study was approved by the Institutional Review Board of Yonsei University, Severance Hospital, Seoul, South Korea (IRB no.4-2016-0421).

### Clinical variables

BMI was defined as subject weight in kilograms (kg) divided by the square of height in meters (m^2^) and was measured at each health examination. Based on baseline BMI, subjects were classified as underweight (BMI less than 18.5 kg/m^2^), normal weight (BMI 18.6–22.9 kg/m^2^), overweight (BMI 23.0–24.9 kg/m^2^), or obese (BMI greater than 25.0 kg/m^2^).[16] Systolic and diastolic blood pressures were also measured at each visit. Serum samples for fasting glucose and total cholesterol were obtained after overnight fasting at each examination visit. A detailed history of smoking status and alcohol consumption were obtained via questionnaire. The latter two measures were categorized for the purpose of this study according to current, former, or never for smoking status and drinker or non-drinker for alcohol intake.

Medical history of participants was identified using a combination of the following: clinical and pharmacy code of the 10^th^ revision of the International Classification of Diseases (ICD-10), lists of prescribed medicine, and previous medical histories. To identify hypertension and diabetic mellitus status, laboratory data were utilized in addition to medical records and health examination survey results based on the criteria of systolic blood pressure (SBP) ≥140 mm Hg or diastolic blood pressure (DBP) ≥90 mm Hg for hypertension and fasting serum glucose ≥126 mg/dL for diabetic mellitus. The laboratory and survey questionnaire data of general and life-transition health examinations for all cohort members were merged.

### BMI change categories

Based on BMI changes from baseline to midpoint follow-up and from midpoint follow-up to final health check, individuals were stratified into five groups as follows:[7] (1) Stable: less than 4% change in BMI from baseline to midpoint to last health checkup; (2) Sustained gain: increased BMI (≥4%) since baseline without BMI decrease (≥4%) during follow-up. This included BMI stability or gain from baseline to midpoint follow-up and further BMI gain from midpoint follow-up to last visit, or BMI gain from baseline to midpoint follow-up followed by stable BMI; (3) Sustained loss: decrease in BMI (≥4%) since baseline without increased BMI (≥4%). This included BMI stability or loss from baseline to midpoint follow-up and further BMI loss from midpoint follow-up to last visit, or BMI loss from baseline to midpoint follow-up followed by stable BMI; (4) Loss-gain: BMI loss from baseline to midpoint follow-up, followed by BMI gain from midpoint follow-up to last visit; and (5) Gain-loss: BMI gain from baseline to midpoint follow-up, followed by BMI loss from midpoint follow-up to last visit. Groups 4 and 5 were included in the category of BMI fluctuation, with no assumptions as to whether such BMI changes were intentional or unintentional.

Mean values and standard deviations (SDs) of BMI at three time points (baseline, midpoint, and last follow-up) were calculated. Direction and magnitude of change in subject’s BMI was determined by regression slope of BMI values (BMI slope). Slopes of SBP, DBP, and total cholesterol were calculated by each regression equation at three time points over time.

### Study endpoint

The study outcomes were death from all causes as well as death from CV and non-CV causes. Mortality status of each participant was ascertained together with specific cause of death. The latter events were classified according to ICD-10 codes that were obtained from the South Korean National Statistical Office.

### Statistical methods

Variables are reported as percentages or as means ± SDs for normally distributed variables and as median (with 25–75% ranges) for non-normally distributed variables, as appropriate. A multivariate Cox proportional hazards regression model was employed to assess the relationship between BMI changes and mortality outcomes by estimating hazard ratios (HR) with 95% confidential intervals (95% CI). To assess the relationships between BMI measures and CV risk factors, we used a multivariate linear regression analysis in which each CV risk factor was treated as the dependent variable, and the independent variables were the baseline value of each CV risk factor of age, smoking status, alcohol consumption, hypertension, and diabetic mellitus. All analyses were conducted using SAS, Version 9.4 (SAS Institute Inc., Cary, NC, USA), with a P value <0.05 considered significant in all analyses.

## Results

Demographic characteristics of the enrolled patients are shown in Table 1. Mean age was 52 ± 9 years, and 56% of the patients were men. Hypertension and diabetic mellitus were observed in 6.3% and 3.3% of the patients, respectively. During a mean follow-up of 10.7 ± 0.9 years (median follow-up 11 years), there were 12,378 (3.3%) deaths from all causes, which consisted of 2,114 (0.6%) CV-related deaths and 10,264 (2.7%) non-CV deaths.

**Table 1:** Characteristics of the study population.

Variables		Total (n = 379,535)	BMI change category					
			Stable (n = 109,805)	Sustained gain (n = 66,021)	Sustained loss (n = 65,783)	Fluctuation		
						All (n = 137,926)	Loss-gain (n = 67,641)	Gain-loss (n = 70,285)
Age		51.7±8.9	51.0±8.4	50.2±8.5	53.4±9.2	52.1±9.1	51.8±8.9	52.3±9.3
Sex	Male	212,653(56.0)	64,842(59.1)	36,273(54.9)	36,777(55.9)	74,761(54.2)	35,599(52.6)	39,162(55.7)
	Female	166,882(44.0)	44,963(41.0)	29,748(45.1)	29,006(44.1)	63,165(45.8)	32,042(47.4)	31,123(44.3)
Hypertension		24,054(6.3)	6,628(6.0)	3,207(4.9)	5,181(7.9)	5,181(7.9)	4,394(6.5)	4,644(6.6)
Diabetes mellitus		12,653(3.3)	3,176(2.9)	1,408(2.1)	3,470(5.3)	4,599(3.3)	2,306(3.4)	2,293(3.3)
BMI (kg/m^2^)		24.0±2.9	24.1±2.7	23.0±2.8	24.8±3.0	23.9±3.0	24.5±3.0	23.4±2.8
BMI category	Normal	135,555(35.7)	36,194(33.0)	32,256(48.9)	16,618(25.3)	50,487(36.6)	20,506(30.3)	29,981(42.7)
	Underweight	7,678(2.0)	1,457(1.3)	2,401(3.6)	720(1.1)	3,100(2.3)	923(1.4)	2,177(3.1)
	Overweight	104,940(27.7)	32,580(29.7)	16,719(25.3)	18,057(27.5)	37,584(27.3)	18,484(27.3)	19,100(27.2)
	Obesity	131,362(34.6)	39,574(36.0)	14,645(22.2)	30,388(46.2)	46,755(33.9)	27,728(41.0)	19,027(27.1)
SBP (mmHg)		126.3±17.7	126.1±17.4	124.0±17.3	128.7±18.2	126.5±17.9	127.2±18.0	125.8±17.8
DBP (mmHg)		79.4±11.6	79.5±11.6	78.3±11.5	80.6±11.8	79.4±11.6	79.8±11.7	79.0±11.5
Fasting glucose (mg/dl)		97.2±32.7	96.2±29.8	94.0±31.9	101.3±35.1	97.5±33.8	98.1±34.6	96.9±33.0
Total cholesterol (mg/dl)		200.3±38.2	200.9±37.8	197.3±37.8	202.9±38.5	200.1±38.3	201.5±38.6	198.7±38.0
Smoking	Never smoker	239,788(66.0)	69,269(65.9)	40,100(63.4)	42,871(68.2)	87,548(66.3)	43,811(67.7)	43,737(64.9)
	Former smoker	33,274(9.2)	10,642(10.1)	5,237(8.3)	5,917(9.4)	11,478(8.7)	5,704(8.8)	5,774(8.6)
	Current smoker	90,284(24.9)	25,224(24.0)	17,884(28.3)	14,080(22.4)	33,096(25.1)	15,237(23.5)	17,859(26.5)
Alcohol consumption	Non-drinker	201,701(54.1)	56,113(52.0)	34,765(53.6)	35,977(55.8)	74,846(55.3)	37,029(55.8)	37,817(54.8)
	Drinker	170,936(45.9)	51,768(48.0)	30,101(46.4)	28,529(44.2)	60,538(44.7)	29,287(44.2)	31,251(45.3)
Mortality								
All-cause		12,378(3.3)	2,998(2.7)	1,564(2.4)	2,901(4.4)	4,915(3.6)	2,220(3.3)	2,695(3.8)
Cardiovascular		2,114(0.6)	533(0.5)	257(0.4)	464(0.7)	860(0.6)	410(0.6)	450(0.6)
Non-cardiovascular		10,264(2.7)	2,465(2.2)	1,307(2.0)	2,437(3.7)	4,055(2.9)	1,810(2.7)	2,245(3.2)

Abbreviations: BMI, body mass index; SBP, systolic blood pressure; DBP, diastolic blood pressure.

Table 2 shows mean BMI measurements across the three time points as well as the mean BMI changes from baseline to last health examination according to BMI change group. The stable group had the lowest mean BMI change, whereas the sustained gain and sustained loss groups had the largest mean BMI changes. Fluctuation groups with increased and then decreased BMI or decreased and then increased BMI showed little overall change in mean BMI.

**Table 2:** Body mass index by weight change category and mean change from baseline to last follow-up.

BMI change category	Mean follow-up duration for BMI change (years)	BMI, kg/m^2^			
		Baseline	Midpoint	Last	Mean change from baseline to last follow-up
Stable	9.3±1.7	24.1±2.7	24.1±2.7	24.1±2.7	-0.01±0.5
Sustained gain	9.5±1.5	23.0±2.8	24.1±2.9	25.2±3.0	2.14±1.1
Sustained loss	9.3±1.6	24.8±3.0	23.7±2.8	22.6±2.8	-2.24±1.3
Fluctuation	9.3±1.6	23.9±3.0	23.9±3.0	23.9±3.0	-0.05±1.5
Loss-gain	9.3±1.6	24.5±3.0	23.0±2.8	24.4±3.0	-0.08±1.5
Gain-loss	9.4±1.6	23.4±2.8	24.8±2.9	23.4±2.9	-0.02±1.5

BMI, body mass index

Table 3 reports the linear regression relationships between BMI variables and each CV risk factor according to BMI category. BMI demonstrated a positive linear relationship with SBP, DBP, and total cholesterol across all categories of BMI change, which remained robust after adjustment for clinical and laboratory covariates.

**Table 3:** Multiple linear regression analysis of the relationships between body mass index variables and cardiovascular risk factors by body mass index change category.

	BMI mean	BMI SD	BMI slope
**Stable**			
SBP slope	0.086 *	0.013 *	0.046 *
DBP slope	0.070 *	0.013 *	0.037 *
TC slope	-0.023 *	-0.005	0.048 *
**Sustained gain**			
SBP slope	0.092 *	0.048 *	0.086 *
DBP slope	0.077 *	0.047 *	0.072 *
TC slope	-0.042 *	0.026 *	0.066 *
**Sustained loss**			
SBP slope	0.085 *	-0.032 *	0.096 *
DBP slope	0.059 *	-0.017 *	0.082 *
TC slope	-0.009 ^§^	-0.056 *	0.099 *
**Fluctuation**			
SBP slope	0.085 *	0.007 ^§^	0.082 *
DBP slope	0.067 *	0.008 ^§^	0.069 *
TC slope	-0.026 *	-0.012 *	0.087 *

All values are standardized multiple regression beta-coefficients (ß) and were adjusted for baseline age, baseline value of systolic blood pressure, diastolic blood pressure, fasting blood sugar, total cholesterol, smoking status, alcohol consumption, hypertension, and diabetic mellitus.

*P <0.001

^§^P <0.05.

Abbreviations: BMI, body mass index; SD, standard deviation; SBP, systolic blood pressure; DBP, diastolic blood pressure; TC, total cholesterol.

Table 4 reports changes in SBP, DBP, and total cholesterol during follow-up according to BMI group. Sustained gain group demonstrated gradual increase of SBP and total cholesterol over follow-up period (P for trend < 0.0001). Contrarily, sustained loss group demonstrated gradual decrease of all those values over follow-up period (P for trend < 0.0001).

**Table 4:** Changes in systolic blood pressure, diastolic blood pressure, and total cholesterol according to BMI group.

Group	Baseline	Mid-point	Last follow-up	P for trend
	Systolic blood pressure			
Stable(n = 109,805)	126.1±17.4	125.7±14.8	125.4±14.9	< .0001
Sustained gain(n = 66,021)	124.0±17.3	125.0±14.7	126.2±15.0	< .0001
Sustained loss(n = 65,783)	128.7±18.2	126.6±15.1	124.7±15.4	< .0001
Fluctuation(n = 137,926)	126.5±17.9	126.0±15.2	125.6±15.3	< .0001
	Diastolic blood pressure			
Stable(n = 109,805)	79.5±11.6	78.3±9.6	77.5±9.8	< .0001
Sustained gain(n = 66,021)	78.3±11.5	78.1±9.5	78.1±9.8	0.0284
Sustained loss(n = 65,783)	80.6±11.8	78.4±9.6	76.6±9.9	< .0001
Fluctuation(n = 137,926)	79.4±11.6	78.3±9.7	77.4±9.9	< .0001
	Total cholesterol			
Stable(n = 109,805)	200.9±37.8	199.8±34.3	198.9±37.2	< .0001
Sustained gain(n = 66,021)	197.3±37.8	200.5±34.6	202.4±38.1	< .0001
Sustained loss(n = 65,783)	202.9±38.5	198.1±35.2	193.1±38.0	< .0001
Fluctuation(n = 137,926)	200.1±38.3	199.2±35.2	198.2±38.4	< .0001

Table 5 reports the relationships between BMI change category and mortality from all causes, CV causes, and non-CV causes. A sustained BMI loss displayed a significantly higher risk of all-cause mortality compared with stable BMI (HR 1.07, 95% CI 1.01–1.13), even after adjustment for clinical variables including the SD of BMI and the mean of BMI. The impact of a sustained BMI loss on CV mortality was neutral, indicating that any increased risk of death was attributable to the increased risk associated with non-CV mortality (HR 1.10, 95% CI 1.04–1.17). Conversely, a sustained BMI gain demonstrated a significantly lower risk of all-cause mortality (HR 0.85, 95% CI 0.79–0.90) compared with stable weight, and this finding was consistent for both CV mortality (HR 0.82, 95% CI 0.70–0.96) and non-CV mortality (HR 0.85, 95% CI 0.79–0.91) after adjustment. BMI fluctuations were associated with an adjusted increase in the risk of all-cause mortality (HR 1.06, 95% CI 1.01–1.11) as well as non-CV mortality (HR 1.07, 95% CI 1.01–1.13); these associations were non-significantly attenuated following adjustment for mean BMI.

**Table 5:** Adjusted hazard ratios for all-cause, cardiovascular, and non-cardiovascular mortality according to weight change category.

BMI change category	Adjusted hazard ratio (95% CI)		
	Model A	Model B	Model C
All-cause mortality			
Stable	1.0 (reference)	1.0 (reference)	1.0 (reference)
Sustained gain	0.89 (0.84–0.95)*	0.81 (0.76–0.86)*	0.89 (0.83–0.95)*
Sustained loss	1.32 (1.26–1.40)*	1.38 (1.31–1.46)*	1.25 (1.19–1.32)*
Fluctuation	1.17 (1.12–1.23)*	1.13 (1.08–1.19)*	1.13 (1.08–1.19)*
Loss-gain(a)	1.11 (1.05–1.18)*	1.13 (1.07–1.19)*	1.06 (1.00–1.13)§
Gain-loss(a)	1.22 (1.16–1.29)*	1.14 (1.08–1.20)*	1.20 (1.13–1.26)*
Cardiovascular mortality			
Stable	1.0 (reference)	1.0 (reference)	1.0 (reference)
Sustained gain	0.84 (0.72–0.98)§	0.79 (0.68–0.92)¶	0.84 (0.72–0.98)§
Sustained loss	1.10 (0.97–1.25)	1.14 (1.00–1.29)	1.06 (0.93–1.20)
Fluctuation	1.10 (0.99–1.23)	1.08 (0.97–1.21)	1.07 (0.96–1.20)
Loss-gain(a)	1.10 (0.96–1.25)	1.10 (0.97–1.26)	1.06 (0.92–1.21)
Gain-loss(a)	1.11 (0.98–1.26)	1.06 (0.93–1.20)	1.09 (0.96–1.24)
Non-cardiovascular mortality			
Stable	1.0 (reference)	1.0 (reference)	1.0 (reference)
Sustained gain	0.90 (0.84–0.96)¶	0.81 (0.76–0.87)*	0.90 (0.84–0.96)¶
Sustained loss	1.38 (1.30–1.46)*	1.44 (1.36–1.53)*	1.30 (1.22–1.38)*
Fluctuation	1.18 (1.12–1.25)*	1.15 (1.09–1.21)*	1.15 (1.09–1.21)*
Loss-gain(a)	1.12 (1.05–1.19)*	1.13 (1.06–1.20)*	1.06 (1.00–1.13)
Gain-loss(a)	1.24 (1.17–1.32)*	1.16 (1.09–1.23)*	1.22 (1.15–1.29)*

BMI, body mass index; Model A was adjusted for age and sex, baseline systolic blood pressure, diastolic blood pressure, serum glucose, total cholesterol, smoking status, alcohol consumption, hypertension, and diabetes mellitus. Model B was adjusted for variables outlined in Model A as well as for baseline BMI. Model C was adjusted for variables outlined in Model A as well as mean BMI. (a) indicates hazard ratios for ‘Loss-gain’ & ‘Gain-loss’ groups versus the ‘Stable’ group when weight change included five categories.

*P <0.001

¶P <0.01

§P <0.05.

**Fig 2:**
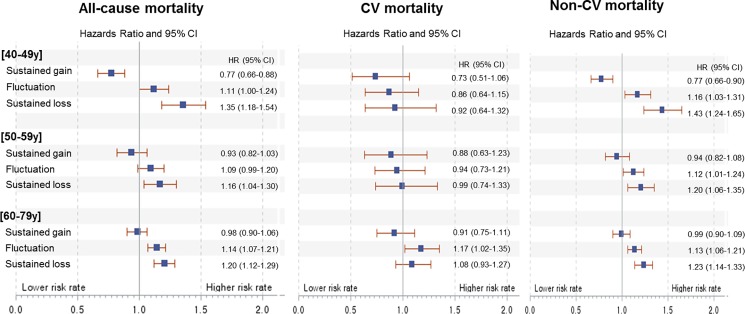
Adjusted hazard ratios for all-cause, cardiovascular, and non-cardiovascular mortality by age. All-cause, CV, and non-CV mortality models were adjusted for age, baseline systolic blood pressure, diastolic blood pressure, serum glucose, total cholesterol, smoking status, alcohol consumption, hypertension, diabetes mellitus, standard deviation of body mass index, and mean body mass index. Abbreviations: CV, cardiovascular; CI, confidential interval; HR, hazard ratio.

We observed no statistically significant interaction between gender and all-cause, CV, or non-CV mortality (all P for interaction >0.05). However, in subgroup analysis of age categories, we found a statistically significant interaction between age and all-cause or non-CV mortality (P for interaction <0.05). Fig 2 demonstrates the association of BMI changes with mortality outcomes according to age. Favorable mortality outcomes for sustained BMI gains groups were prominent in persons between ages of 40 and 49. Sustained BMI gains in those aged 40–49 showed the lowest mortality risk among the four groups; that is, all-cause mortality was lower in patients with sustained BMI gains compared with those showing stable BMI (HR 0.78, 95% CI 0.72–0.84). However, the benefit of sustained BMI gain was slightly attenuated in patients with age over 50; sustained BMI gain and stable BMI demonstrated similar outcomes, both of which were more favorable than BMI loss and fluctuation. The sustained BMI gain was associated with comparable all-cause mortality to that of stable BMI in individuals aged 50–59 (HR 0.93, 95% CI 0.82–1.03) and in those aged 60–79 (HR 0.98, 95% CI 0.90–1.06).

## Discussion

The principal findings of the current study were (1) BMI gains in middle-aged adults were associated with a lower risk of all-cause, CV, and non-CV mortality; (2) conversely, BMI loss and fluctuation were associated with increased mortality risk, which was mainly attributable to an increase in the rate of non-CV mortality.

### BMI change and survival

The potential detrimental effects of BMI loss have been substantially reported.[17–19] weight loss has long been associated with increased mortality and increased risk of hip fracture, irrespective of current weight or intention to lose weight. In the current study, BMI loss was predominantly associated with an increased risk of non-CV mortality, which is somewhat concordant with previous studies.

To date, however, the harmful or beneficial impact of BMI gain on mortality in middle-aged adults has received little attention, particularly beyond Western societies. There are conflicting results on this topic. Some have reported that weight gain up to midlife is considered to impair quality of health in later life,[20] and childhood obesity is associated with premature mortality.[21,22] In contrast, the present study indicated that a mild weight gain, reflected by BMI gain, might actually reduce the rate of all-cause mortality, which is somewhat consistent with the few previous studies showing better outcomes with BMI gain.[7,12]^,^[23]

A novel finding of the current study was that BMI gains beyond midlife might actually associated to a more favorable prognosis, despite the finding that the baseline prevalence of obesity and overweight in the weight gain group was reasonably high at 25% and 22%, respectively. Notably, the favorable outcome on all-cause mortality was not solely attributable to a decline in non-CV mortality. Instead, the beneficial impact of a sustained BMI gain on all-cause mortality appeared to reflect reduction in both CV and non-CV mortality in the sustained BMI gain group, which contradicts our expectation that CV mortality risk would most likely increase on the background of BMI gain. Clearly, this warrants further investigation. Interestingly, BMI loss was associated with unfavorable outcomes, even after adjusting for baseline or mean BMI. The overall prevalence of obesity or overweight at baseline was higher in the BMI loss group, in comparison to BMI gain group or stable BMI group. Therefore, in contrast to our perception that BMI loss would be helpful for those with overweight or obesity, impact of BMI loss on clinical outcomes needs to be further investigated in healthy middle-aged persons, even when they are overweight or obese.

### Metabolically healthy obesity

One plausible explanation for improved outcomes associated with BMI gains might be linked to the term ‘metabolically healthy obesity,’ which was generated to describe the absence of components of metabolic complications in the presence of increased BMI.[24] The study population demonstrated a low prevalence of chronic conditions such as hypertension and diabetic mellitus and presented with a nearly normal range of laboratory tests and blood pressure at baseline examination. Intriguingly, improved clinical outcomes were observed in the sustained BMI gain group, even though BMI gains were associated with increased levels of CV risk factors including blood pressure and total cholesterol in this group. To this end, we speculate that any BMI gains observed in the metabolically healthy individuals might have conferred a protective effect against the risk of CV and non-CV mortality. This finding is somewhat thought provoking considering that BMI gain itself typically induces an increased presence of CV risk factors such as blood pressure or cholesterol, as found in the current study, according to the linear relationships between BMI and other CV factors, as well as an increase in systolic blood pressure and total cholesterol during follow-up in BMI gain group. This might, in part, be associated with the notion that incidence of coronary heart disease is often not increased in metabolically healthy obese persons [25], and high BMI may survive longer when compared to their counterparts with lower BMI, although high BMI is associated with higher prevalence of diabetic mellitus and CV disease. Also, heart failure [26] and stroke [27], as well as coronary heart disease, have demonstrated this obesity paradox regarding survival, and these factors might have influenced the results of this study in light of the lower rates of all-cause and CV mortality observed in the sustained BMI gain group.

### Change in body composition after midlife

Another possible explanation for the favorable impact of BMI gain on prognosis might be the change in body composition that is often observed during the latter stages of the aging process. Body weight gradually increases throughout adulthood until approximately 60 years of age, after which time the mean body weight appears to decrease.[28] Most notably, lean body mass, predominantly muscle, is lost at an accelerated rate, with and without intentional weight loss, as individuals age.[29] Stable weight individuals who maintain adult weight through their 6^th^ and 7^th^ decades are not necessarily protected from age-related muscle loss;[30] this has been reported through a substantial shift in body composition marked by progression of sarcopenia across a 5-year period in healthy, ambulatory, stable weight elderly subjects.[31] Newman et al. also reported that lean body mass decreased in stable weight patients with increasing age, but increased among those who gained weight, although it should be noted that fat mass gain was still considerably higher in that study.[32]

Lean body mass could therefore be a feasible explanation for why BMI loss carries a higher mortality risk, whereas BMI gain might be associated with a better clinical prognosis. Considering a report that mortality was highest in coronary heart disease patients with both low body fat and low lean mass and lowest among those with high fat and high lean mass,[19] maintaining lean body mass through subtle BMI gains during mid-to-late life might prove critical for better health in older individuals, even though mechanism for explanation needs further investigation regarding theories stating that BMI gain might provide a safer storage location for environmental toxins.[33] If proven in forthcoming studies, preserving the concept of “healthy age-related mild weight gains” to maintain lean body mass in order to reduce adverse outcomes should be advocated following midlife. This might also result in the most favorable clinical outcomes in BMI gain individuals, who might not lose lean body mass during the aging process.

### Limitations

An inherent limitation of this study includes a potential selection bias. The NHIS recommends, but does not obligate all health insurance subscribers to undergo biennial health examinations. Hence, this cohort probably consisted of healthier individuals or those who are more concerned about their health. However, the NHIS-HEALS data reflect a representative sample of Koreans who were prospectively followed during the study period. Additionally, the participation rate of the general health screening program was high: up to 74.8% in 2014 according to NHIS.[34] We cannot discount the possibility that the current findings might have differed if we used information on other more robust measures of obesity including body composition, visceral fat mass,[35] and waist circumference. Amounts of drinking and smoking and menopause status in women influence BMI changes and mortality. Further studies would be helpful to evaluate the interactions among these factors. In this study, we used BMI rather than weight to assess body weight changes, since our dataset lacks information regarding body weight and height, and only includes BMI value calculated from weight and height. Since BMI could be affected by both weight and height, we cannot exclude the possibility that changes in height and weight could affect BMI changes, although height change might be relatively smaller than weight change. It would be helpful to look at the prevalence of metabolic syndrome among the groups. However, we do not have information regarding waist circumference, HDL-cholesterol and triglyceride. Further studies regarding association between moderate BMI gain and changes in prevalence of metabolic syndrome is warranted. BMI change groups were defined by percent changes, which could be affected by the initial body weight of the individual. However, as there is no universal definition of BMI change groups, we followed criteria of a previous study.[7] The lack of reliable information related to intentional and unintentional weight loss or gain further limits interpretation of the present study findings. BMI change, as well as events and changes of CV risk factors, were all assessed during the same follow-up period, which can be biased. If we had performed analysis for BMI change in a certain period and then performed clinical follow-up thereafter, different formulations might have led to different data analytic results. However, as our main focus was to investigate the way BMI changes as people go toward event, we decided to perform the concurrent analysis of BMI changes and events. Although we analyzed BMI at three separate time points over the course of the study follow-up period, BMI variability between examinations was not assessed. Future studies should provide additional insight by integrating a prospective and possibly time-dependent assessment of the relationship between BMI-associated weight gain and risk of poor outcome in middle-aged adults.

## Conclusion

Sustained BMI gains were associated with decreased risk of all-cause, CV, and non-CV mortality in middle-aged adults. Our current findings support the notion that mild or moderate BMI gain during mid-to-late life is not harmful in metabolically healthy persons, but is rather associated with a favorable outcome in comparison to BMI loss and fluctuation, and even to stable BMI.
